# It is really time to retire laparoscopic gastric banding? Positive outcomes after long-term follow-up: the management is the key

**DOI:** 10.1007/s13304-021-01178-1

**Published:** 2021-10-01

**Authors:** Francesco Saverio Lucido, Giuseppe Scognamiglio, Giusiana Nesta, Gianmattia del Genio, Stefano Cristiano, Francesco Pizza, Salvatore Tolone, Luigi Brusciano, Simona Parisi, Stefano Pagnotta, Claudio Gambardella

**Affiliations:** 1 Division of General, Mininvasive and Bariatric Surgery, University of Campania “Luigi Vanvitelli”, Via Pansini 5, 80100 Naples, Italy; 2 General and Bariatric Surgery Unit, Camilliani Hospital, Casoria, Italy; 3 Department of Surgery, Hospital “A. Rizzoli”, Lacco Ameno, Naples, Italy; 4 Department of Science and Technology, University of Sannio, 82100 Benevento, Italy

**Keywords:** Laparoscopic adjustable gastric banding, Weight regain, Slippage, Laparoscopic sleeve gastrectomy

## Abstract

After the initial widespread diffusion, laparoscopic adjustable gastric banding (LAGB) has been progressively abandoned and laparoscopic sleeve gastrectomy (LSG) has become the worldwide most adopted procedure. Nevertheless, recent reports raised concerns about the long-term weight regain after different bariatric techniques. Considering the large LAGB series recorded in our multicentric bariatric database, we analysed the anthropometric and surgical outcomes of obese patients underwent LAGB at a long-term follow-up, focusing on LAGB management. Between January 2008 to January 2018, demographics, anthropometric and post-operative data of obese patients undergone LAGB were retrospectively evaluated. To compare the postoperative outcomes, the cohort was divided in two groups according to the quantity of band filling (QBF): low band filling group (Group 1) with at most 3 ml of QBF, and patients in the high band filling group (Group 2) with at least 4 ml. 699 obese patients were considered in the analysis (351 in Group 1 and 348 in Group 2). Patients in Group 1 resulted significantly associated (*p* < 0.05) to higher % EWL and quality of life score (BAROS Score), 49.1 ± 11.3 vs 38.2 ± 14.2 and 5.9 ± 1.8 vs 3.8 ± 2.5, respectively. Moreover, patients with lower band filling (Group 1) complained less episodes of vomiting, epigastric pain and post-prandial reflux and significantly decreased slippage and migration rate (*p* < 0.001 for all parameters). LAGB is a safe and reversible procedure, whose efficacy is primarily related to correct postoperative handling. Low band filling and strict follow-up seem the success’ key of this technique, which deserves full consideration among bariatric procedures.

## Introduction

After the initial success, related to its satisfactory short-term weight loss, low perioperative mortality and reversibility, in the last 20 years, we assisted to a decline in the use of laparoscopic adjustable gastric banding (LAGB), reaching nowadays only 5% of all bariatric surgeries [1–5]. Long-term weight regain (WR) determining the frequent occurrence of revisional surgery and the not negligible postoperative complications (i.e., slippage, migration, erosion) remain some of the major concerns about LAGB. Therefore, the bariatric community progressively reduced and abandoned this technique [5–8].

Conversely, we assisted to the widespread of different bariatric procedures, such as Roux-en-Y gastric bypass (RYGB) (38.6%) and laparoscopic sleeve gastrectomy (LSG) (46.0%), since the latter has become the first bariatric surgical procedure worldwide performed [5, 9, 10].

Regarding the restrictive procedures, prospective and retrospective studies largely demonstrated the advantages of LSG including the relative easiness of the technique, the short operative time and the immediate caloric intake restriction [9–11]. Nevertheless, along with the multitude of surprising reports about the efficacy in terms of rapid percentage of excess weight loss (% EWL) after LSG, numerous recent studies raised concerns about long-term results [12–14]. Surely the large diffusion of this procedure has consequently increased the number of patients undergoing revision surgery after LSG for the occurrence of insufficient % EWL or WR at long-term follow-up, representing a confounding factor [15, 16].

In our experience, as for the global bariatric community, gastric banding has followed a descending trend. In the last 5 years, in fact, LAGB was almost totally replaced by LSG [9, 10]. However, considering the large series of LAGB previously performed in our bariatric departments and recorded in our multicentric database, we aimed to analyse the anthropometric and surgical outcomes of a large cohort of obese patients who underwent LAGB at a long-term follow-up. We focused on calibration of gastric banding during follow-up and its relationship with weight loss and onset of postoperative complications. Aim of this retrospective multicentric study is to highlight the importance of an appropriate management of LAGB analysing the correlation between the fluctuation of postoperative anthropometric features, of postoperative complications and of the onset of most common symptoms after LAGB positioning at different quantities of band filling.

## Methods

### Study design

A retrospective multicentric study comparing the postoperative complications, the anthropologic outcomes and the quality of life in patients undergoing LAGB were performed. This study is reported according to the Strengthening the Reporting of Observational Studies in Epidemiology (STROBE) statement for cohort studies [17].

### Study setting and study population

Between January 2008 to January 2018, consecutive adult obese patients undergone LAGB implantation at the University of Study of Campania “Luigi Vanvitelli” of Naples and “Camilliani” Hospital of Casoria (Naples) were retrospectively analysed.

Patient inclusion criteria were as follows: patients aged between 18 and 65 years, affected for at least 5 years by morbid obesity (BMI > 40 or > 35 with co-morbidities) with transient or insufficient response to nutritional treatment, according to the International Federation for Surgery of Obesity (IFSO) guidelines [18]. Patients with a diagnosis of bulimia nervosa or binge eating disorder according to the DSM-IV were excluded from our study. Presence of pre-existing hiatus hernia (HH) greater than 2 cm, with a diagnosis of GERD based on the presence of typical symptoms and evidence of esophagitis (even Grade A of Classification of Los Angeles) at esophagogastroduodenoscopy (EGDS) were considered exclusion criteria. Moreover, patients undergoing abdominal surgical interventions or major emergency operations not related to LAGB, were not considered in the current study to exclude confounding factors that could affect patients ‘symptoms perception.

### Pre-operative evaluation

All patients were referred to bariatric surgery after unsuccessful non-surgical management (i.e., sport activity, dietary restriction). Before surgery, all patients underwent laboratory tests (blood cells count, electrolytes panel, urinalysis, thyroid hormones tests, sex hormones evaluation), chest X-ray, ECG examination, bilateral lower limbs color doppler and cardiological counselling. Moreover, an EGDS, to exclude any pathological conditions, and a psychiatric interview, to evaluate the patient’s motivation, were performed.

The patient’s preoperative work-up was completed by abdominal ultrasound scan, an endocrinological evaluation and an anaesthesiologic scoring.

In all cases, during the preoperative period, both antibiotic prophylaxis (premedication with 2 g of cefazolin i.v.) and antithrombotic prevention (standard dosage regimen of low-molecular-weight heparin LMWH) were adopted.

### Surgical technique

All interventions were performed laparoscopically by three experienced bariatric surgeons (over 200 bariatric procedures performed). The four-trocar technique was used in each procedure.

Pneumoperitoneum was obtained by bladed optical access trocar (VisiPort™ Plus). The left liver lobe was retracted upward, and the lesser curvature of the stomach (pars flaccida) was incised at the point planned for the location of the band. The right crus of the diaphragm was identified. Then a gentle posterior blunt dissection between the crus and the gastric wall was performed.

The grasper was inserted posteriorly to the gastroesophageal junction, reaching the angle of His and carefully avoiding the esophagus, the stomach, the spleen, the diaphragm and the left gastric vessels and the short gastric vessels.

The band was introduced into the abdomen through a 15-mm trocar and was passed posteriorly to the stomach through the opening on the lesser curvature. The band-end tags were then locked and the access port was connected and fixed. Sterile saline was injected to test the correct device functioning.

After the band was locked a few gastric-gastric stitches used to secure the band at the upper part of the fundus, nearby to the left crus. In all cases, we did not adopt the gastric plication.

Finally, the inflation port was fixed through direct suture in mesogastric area, tunnelling from left subcostal trocar.

In the current series, Allergan (LAP-BAND-AP^®^ System surgery) and Helioscope (HELIOGAST^®^ HAGE) gastric banding devices were used, both reaching as the maximum quantity of band filling (QBF) 10 ml.

### Outcome measures

Gender, age, preoperative body mass index (BMI), postoperative complications, and % EWL were retrospectively collected. The operative time was recorded in minutes. The length of stay was evaluated in days. During the hospitalization, the day after surgery, an X-ray with hydro-soluble contrast agent (Gastrographin) was performed in all patients to evaluate the correct location of the band and to exclude erosion or leakage. At discharge, every patient received nutritional support and dietary instructions starting from a liquid diet in the first 3 days to reach a soft diet for the first weeks. Proton pump inhibitor (PPI) was administered after surgery for one month, regardless of the MRGE symptoms occurrence.

Postoperative outpatient controls were performed 2 weeks after surgery and subsequently quarterly for one year. Additional outpatients’ visits were planned in correlation with the level of weight loss and the level of satiety through a Visual Analogue Scale (0–10), ranging from “I am not hungry at all” and “Very hungry” [19]. During the outpatient visits, an accurate clinical evaluation comprising the collection of data about patient’ anthropometric features (weight, BMI, % EWL) was performed. Patients were interviewed about any postoperative discomfort and the eventual onset of symptoms such as dysphagia, reflux, food intolerance or vomiting episodes, and administered therapy were recorded. The QBF (in millilitres) was recorded in all patients, during the outpatient visits. The computation of cumulative band filling was based on saline added/subtracted at every adjustment. Every adjustment was performed of 1 millilitre based on the patients’ satiety level. In case of the onset of postoperative symptoms (i.e., dysphagia, reflux, food intolerance or vomiting), and according to the patients’ tolerance, the device was adjusted or totally deflated. Moreover, routine fluoroscopy examination with the use of oral contrast and an EGDS were performed yearly or in case of the onset of the abovementioned symptoms. If any postoperative complications (i.e., slippage, migration) occurred the LAGB was totally deflated as well.

To analyse the patients’ quality of life, we adopted the Bariatric Analysis and Reporting System (BAROS) Score, introduced by Oria and Moorehead in 1997, which was administered to all patients during outpatient visits. This system is based on a scoring table applied to three main areas (% EWL, modifications in medical conditions, and quality of life assessment), that adds or subtracts points according to positive or negative outcomes, respectively [20].

### Study outcomes

The first endpoint of the study was to evaluate the correlation between the fluctuation of postoperative anthropometric features (BMI, % EWL and BAROS Score) at different QBFs. The secondary endpoint aimed to assess the onset of postoperative complications (i.e. slippage, erosion, migration) and of the most common symptoms after LAGB positioning (post-prandial reflux, epigastric pain, food intolerance and episodes of vomiting) at different QBFs.

### Statistical analysis

Sample size was calculated setting a power of 0.90 for quantitative variables (i.e. % EWL), assuming the hypothesis of 20% parameters improvement following LAGB implantation. To reach a significance set at *p* < 0.05 for clinical, enrolment of ≥ 46 patients was needed.

Data analysis has been performed with R version 3.6.2 [21]. Continuous data are expressed as mean and Standard Deviation (SD) unless otherwise indicated and categorical variables as frequencies and percentages. For all tests, a two-sided *p* < 0.05 was considered statistically significant. The % EWL and BAROS Score were considered the responses of the linear models where the other variables acted as predictors. First, a full linear model was designed involving all the predictors; then, those predictors having a coefficient with a p-value less than 0.01 were removed. The restricted linear models have been estimated from scratch.

The variable QBF was adopted to divide and compare the postoperative outcomes in our cohort. To obtain an optimal dichotomization we split the cohort according to three rules of QBF analysing which was the best to analyse the response variables (% EWL and BAROS Score) in subsets maximally separated and with the lowest within-group variability. The rules were as follows: R1 (QBF = 0, 1, and 2 or QBF > 2), *R*^2^ (QBF = 0, 1,2, 3, or QBF > 3), R3 (QBF = 0,1,2,3 and 4, or QBF > 4). For each of the abovementioned rules, and each of the response variables, an *R*^2^ ANOVA analysis was applied. The *p* values resulted in each case over 2.2 × 10^–16^. We also followed the analysis using the Pearson’s ratio correlation. To evaluate the association between exposure to high band filling and postoperative complications and events, an Odds ratio (OR) analysis was performed.

## Results

**Fig. 1:**
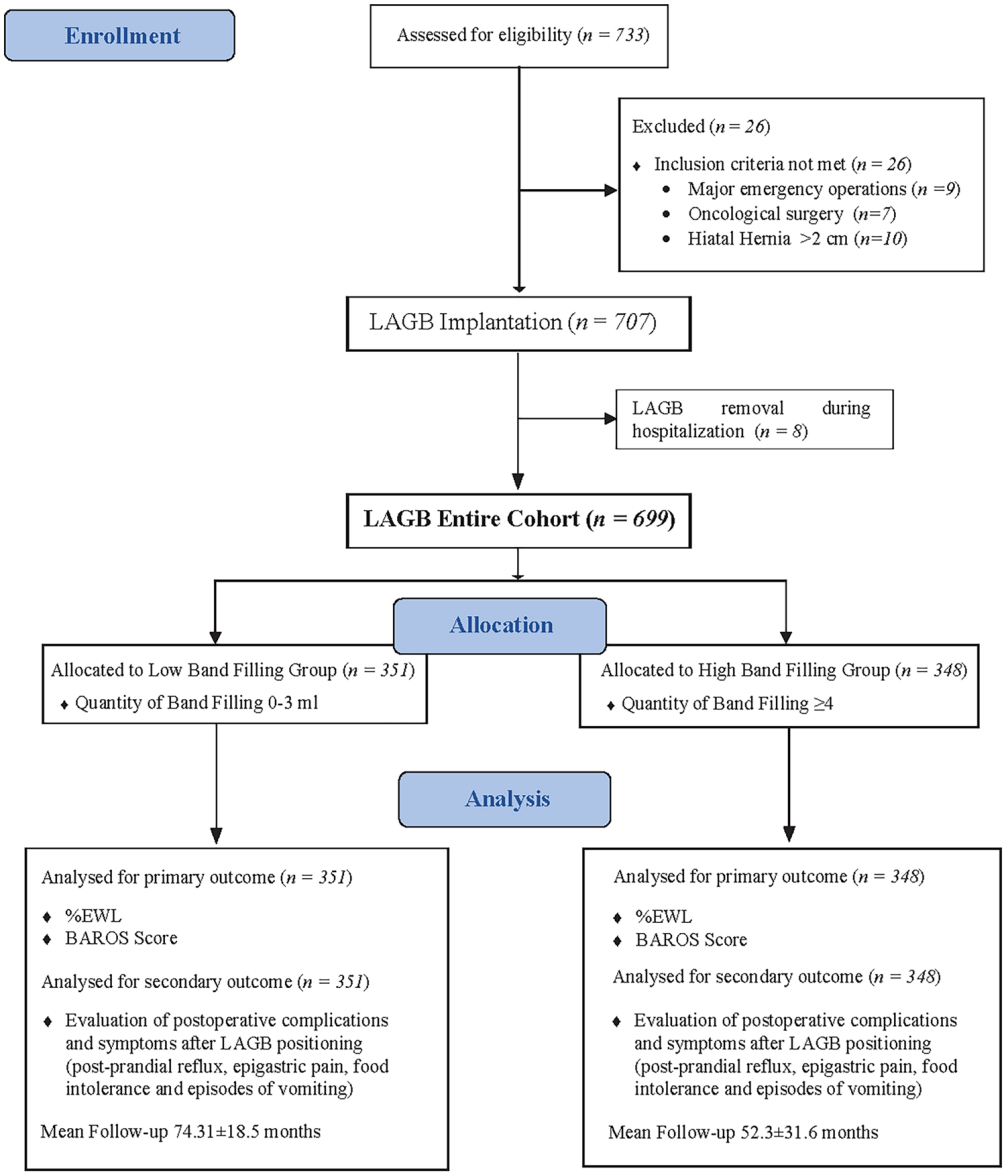
Study design. *% EWL* percentage of excess weight loss, *LAGB* laparoscopic adjustable band filling

Seven hundred and thirty-three patients referred for LAGB implantation for morbid obesity from January 2008 to January 2018 and seven hundred and seven received the procedure. Eight patients were not considered in the analysis having no follow-up for rapid intolerance to LAGB requiring its removal during the hospitalization (3 in postoperative day 2 and 5 in postoperative day 3). The working database counted 699 subjects; 601 were female (86%) (Fig. 1). The baseline demographic characteristics of the study population, recorded 2 weeks before surgery, were detailed in Table 1. The mean age was 30.5 ± 7.8 with a preoperative mean BMI 41.4 ± 3.2.

**Table 1:** Descriptive postoperative statistics of the entire cohort

	699 patients
Postoperative BMI	33.3 ± 7.1
Postoperative % EWL	42.5 ± 11.5^a^
Vomiting	222 (31.7%)
Post-prandial reflux	299 (42.7%)
Epigastric pain	295 (42.2%)
Food intolerance	235 (33.6%)
LAGB migration	31 (44.3%)
LAGB slippage	61 (8.7%)
LAGB removal	167 (23.9%)
Redo surgery	57 (8.2%)
Quantity of band filling (ml)	4.2 ± 2.5^a^
Postoperative BAROS Score	4.9 ± 2.2^a^
Follow-up length (months)	64.1 ± 26.9^a^

*BMI* body mass index, *% EWL* percentage of excess weight loss, *LAGB* laparoscopic adjustable gastric banding

^a^Values are mean ± SD

Mean operative time was 52 min (range 37–69 min). In 379 cases a LAP-BAND-AP^®^ was implanted, while a HELIOGAST^®^ device was adopted in the remaining 320 subjects. No conversion to open surgery was performed. After surgery, the device was left deflated. In two cases a splenic haemorrhage occurred, appropriately laparoscopically treated with electrocoagulation and haemostatic agents. No severe peri-operative complications occurred. Patients ambulated 1 day after surgery. Mean time of hospitalization was 2.3 ± 1.9 days.

At a mean follow-up of 64.1 ± 26.9 months, BMI resulted 33.3 ± 7.1, EWL% percentage of the entire cohort (*n* = 699) was 42.5 ± 11.5, with a mean BAROS Score of 4.9 ± 2.2. No mortality was reported. (Table 1). According to the routine use of fluoroscopy with oral contrast, sixty-one patients (8.7%) developed pouch dilatation and LAGB slippage. Overall mean quantity of postoperative band regulation was 4.2 ± 2.5 ml and the first band filling was done at least one month after surgery in all patients. Each band regulation corresponded to 1 ml of saline solution.

Focusing on the treatment of postoperative events, in all the slippage cases a surgical approach was adopted. Among 61 patients who developed slippage, in 59 patients the band was completely removed (96%). Only in 2 patients the band was repositioned (4%). Migration as well was always surgically approached, requiring LAGB removal in all cases (31/31, 100%).

In the current study, 15 out of 699 (2.4%) patients declined to answer to the Quality of Life Questionnaire (BAROS Score).

### Patients’ dichotomization

To analyse and compare the correlation between band filling and the anthropometric outcomes, the cohort was divided into two groups. First, we separated the patients having at most 2 ml of band filling (245 patients out of 699), and those having 3 ml or more. The R^2^ ANOVA respect to % EWL and BAROS Score resulted 9.92%. Subsequently, we tried to split the cohort according to the other rules: “at most 3 ml, and at least 4 ml”, and “at most 4 ml, and at least 5 ml”. In both cases we computed the *R*^2^ ANOVA reaching 16.35% (with 351 patients out of 699) and 17.71% (with 405 patients out of 699), respectively. The higher gain in *R*^2^ for % EWL and BAROS Score was between the splits “at most 2 ml” and “at most 3 ml”. Therefore, we decided to consider 3 ml of QBF as the cut-off to separate the cohort. The results were detailed in Table 2.

**Table 2:** *R*^2^ ANOVA for % EWL and BAROS Score at different threshold value of band filling

	Threshold value = 2 ml	Threshold value = 3 ml	Threshold value = 4 ml
% EWL	19.22	25.08	25.75
BAROS Score	9.68	16.05	17.40
Linear combination	9.92	16.35	17.71

*% EWL* percentage of excess weight loss

Therefore, 351 patients were allocated in the low band filling group (Group 1) with at most 3 ml of QBF, and 348 patients in the high band filling group (Group 2) with at least 4 ml. The patients in Group 1 received 185 LAP-BAND-AP^®^ and 166 HELIOGAST^®^, while in Group 2, 194 subjects received LAP-BAND-AP^®^ and 154 HELIOGAST^®^ (*p* = 0.419).

**Fig. 2:**
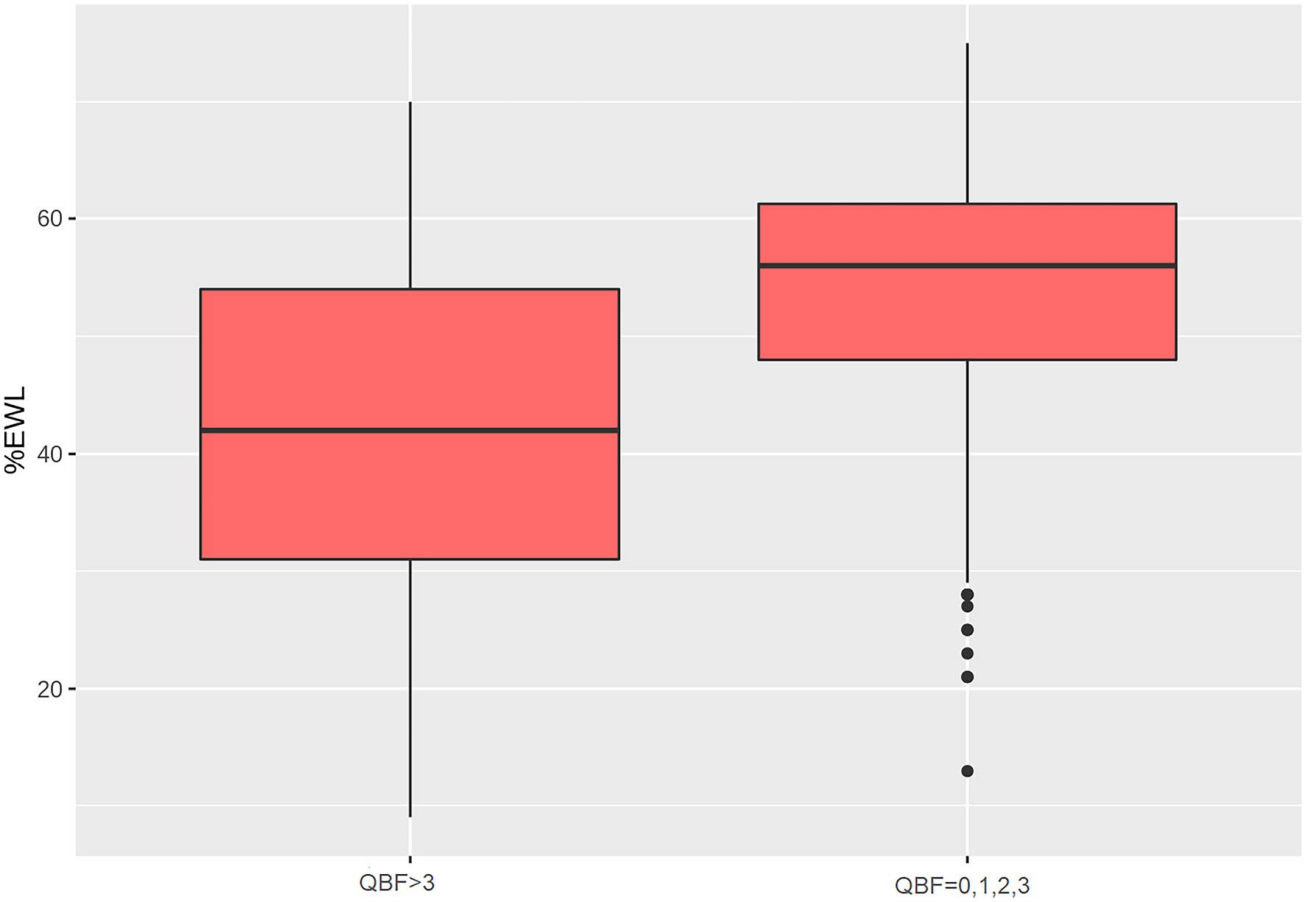
Boxplots displaying the percentage of excess weight loss in case of none, 1, 2, or 3 ml of band filling (right), compared to patients with 4 ml or more band filling (left)

The mean difference of % EWL between patients receiving at most 3 ml of QBF and those with 4 ml or more was significant with a *p* value < 0.01 according to Welch's t-test. Moreover, the bound 3 leads also to a more definite separation of the cohort according to the BAROS Score. Figure 2 shows the differences of the two sub-populations with a *p* value < 0.01 according to Welch's *t* test and an *R*^2^ ANOVA equals to 25.55% (Table 3).

**Table 3:** Postoperative anthropometric outcomes, symptoms and events in low band filling group (Group 1), and in high band filling group (Group 2)

	Group 1–351 patients (QBF ≤ 3 ml)	Group 2–348 patients (QBF ≥ 4 ml)	*p*
BMI^a^	30.3 ± 2.8	35.8 ± 3.7	< 0.05
% EWL^a^	49.1 ± 11.3	38.2 ± 14.2	< 0.05
BAROS Score^a^	5.9 ± 1.8	3.8 ± 2.5	< 0.05
Vomiting	16 (4.6%)	206 (59.2%)	< 0.001
Post-prandial reflux	56 (15.9%)	243 (69.8%)	< 0.001
Epigastric pain	85 (24.2%)	210 (60.3%)	< 0.001
Food intolerance	92 (26.2%)	143 (19.2%)	< 0.001
LAGB migration	1 (0.3%)	30 (8.6%)	< 0.001
LAGB slippage	1 (0.3%)	60 (17.2%)	< 0.001
LAGB removal	12 (3.4%)	155 (44.5%)	< 0.001
LAGB partial deflation	20 (5.7%)	100 (28.7%)	< 0.001
LAGB total deflation	5 (1.4%)	105 (30.2%)	< 0.001
Redo surgery	5 (1.4%)	52 (14.9%)	< 0.001
LAGB follow-up^a^	74.3 ± 18.5	52.3 ± 31.6	< 0.05

*QBF* quantity of band filling, *% EWL* percentage of excess weight loss, *LAGB* laparoscopic adjustable gastric banding)

^a^Values are mean ± SD

### Primary outcome

**Fig. 3:**
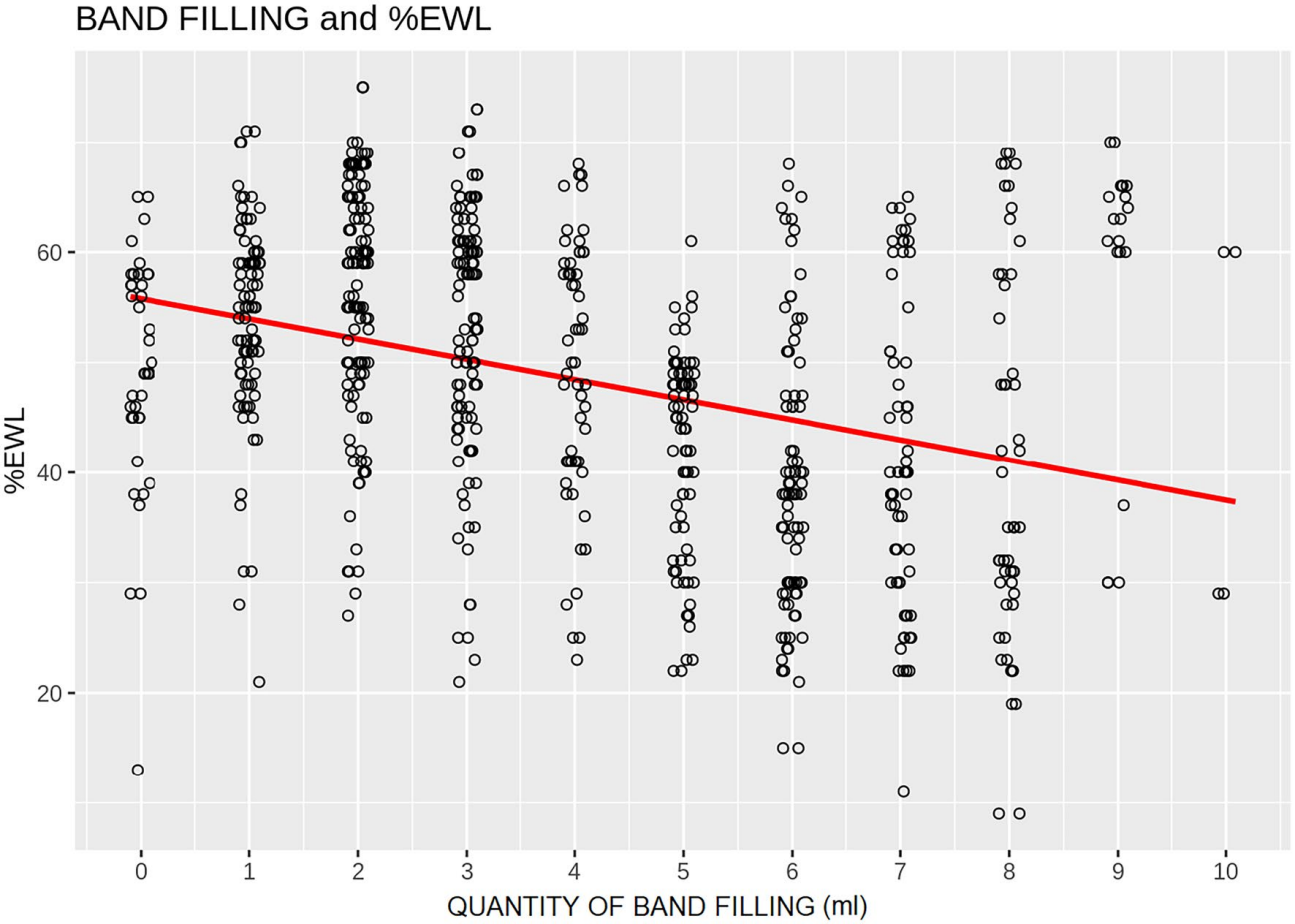
Scatter diagram between quantity of band fillings versus EWL%; both variables are jittered to appreciate the density of patients. As the quantity of band filling increases, the % EWL decreases, starting from about 56% and reaching 37% after 10 ml of band filling. The red line (OLS estimate of regression line) shows the trend. Its slope is − 1.8 (*p* value < 0.01)

In Group 1, after a mean follow-up of 74.3 ± 18.5 months, patients presented a postoperative BMI of 30.3 ± 2.8 experiencing a % EWL of 49.1 ± 11.3 and a BAROS Score of 5.9 ± 1.8. In Group 2, after a mean follow-up of 52.3 ± 31.6 months, patients showed a BMI of 35.8 ± 3.7, % EWL of 38.2 ± 14.2 and a BAROS Score of 3.8 ± 2.5. The difference among % EWL, BAROS Score and length of follow-up in Group 1 and Group 2 resulted statistically different (*p* < 0.05) (Table 1). Figure 3 shows the scatter of the association between QBF and % EWL.

### Secondary outcome

**Fig. 4:**
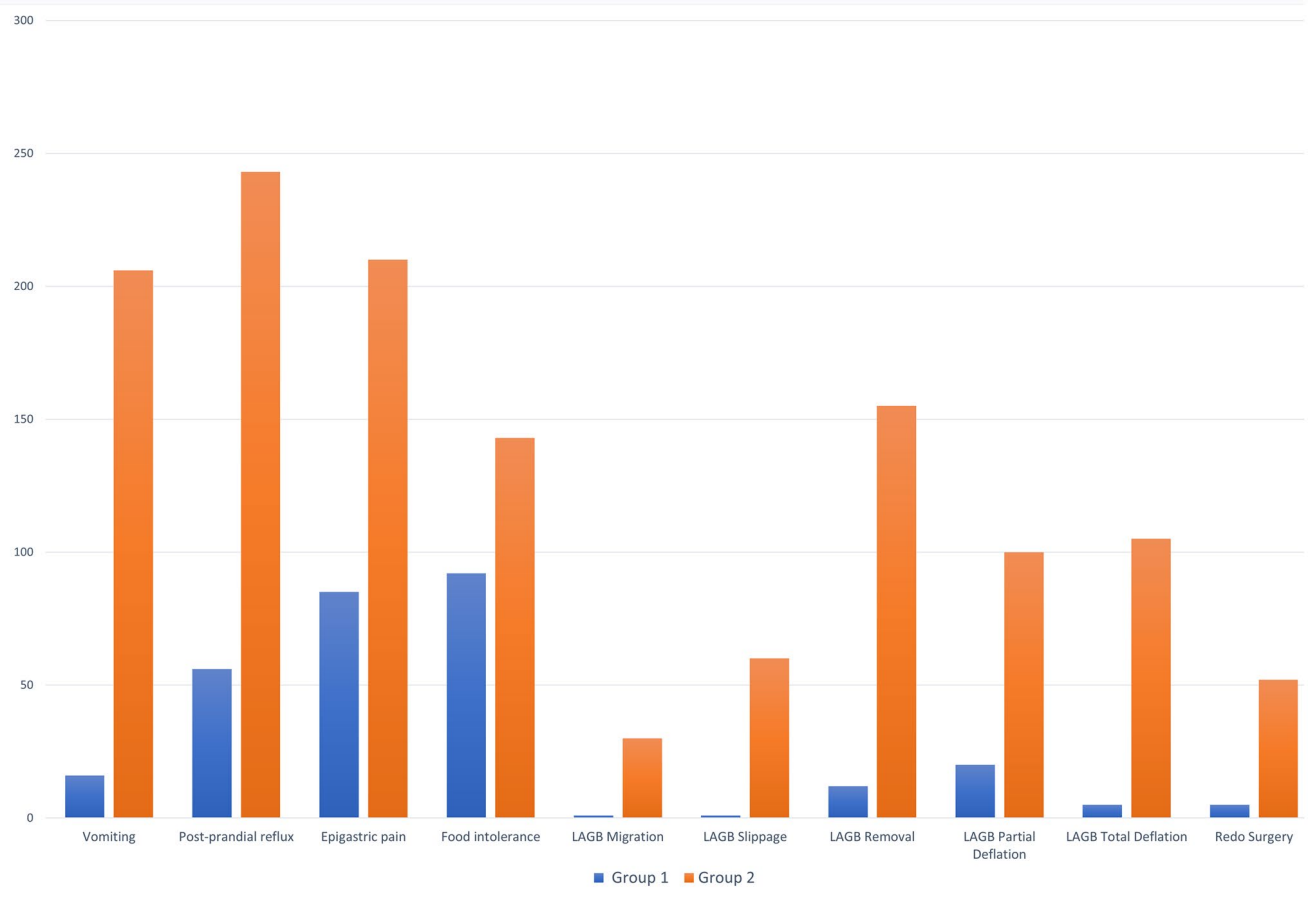
Barplots displaying the percentage of postoperative symptoms and events in Group 1 and Group 2

Regarding postoperative symptoms and events, patients in Group 1 experienced lower rate of symptoms as vomiting (16 vs 206, *p* < 0.001), postprandial reflux (56 vs 243, *p* < 0.001), epigastric pain (85 vs 210, *p* < 0.001) and food intolerance (92 vs 143, *p* < 0.001); similarly, postoperative events such as LAGB migration (1 vs 30), LAGB slippage (1 vs 60), LAGB removal (12 vs 155) and number of bariatric reinterventions (5 vs 52), resulted all significantly lower in Group 1 (*p* < 0.001). Therefore, high band filling resulted significantly associated (*p* < 0.001), to postoperative vomiting, post-prandial reflux, epigastric pain, food intolerance, LAGB migration, LAGB slippage, LAGB removal and further bariatric surgery with an OR of 30.37, 12.19, 4.76, 1.96, 33.02, 72.92, 22.69, 12.16, respectively. (Table 4; Fig. 4) Regarding device deflation, 20 patients of Group 1 experienced a partial deflation vs 100 patients in Group 2 (*p* < 0.0001), while a total deflation occurred in 5 patients of Group 1 and 105 patients of Group 2 (*p* < 0.0001).

**Table 4:** Odds ratio of the association of high band filling patients (Group 2, 348 subjects) with postoperative complications and events

	Odds ratio	95% CI	*p*
Vomiting	30.37	17.61–52.40	< 0.001
Post-prandial reflux	12.19	8.45–17.58	< 0.001
Epigastric pain	4.76	3.44–6.59	< 0.001
Food intolerance	1.96	1.43–2.70	< 0.001
LAGB migration	33.02	4.48–243.53	< 0.001
LAGB slippage	72.92	10.04–529.39	< 0.001
LAGB removal	22.69	12.29–41.89	< 0.001
Redo surgery	12.16	4.79–30.83	< 0.001

*LAGB* laparoscopic adjustable gastric banding

In Group 1, the causes of LAGB removal were as follows: 1 for slippage, 1 for migration, 5 for the performing of a further bariatric procedure, 3 for food intolerance and 2 for pregnancy. In Group 2, the causes of LAGB removal were as follows: 60 for slippage, 30 for migration, 52 for the performing of a further bariatric procedure, 4 for food intolerance, 4 for vomiting and 5 for pregnancy.

## Discussion

In the last twenty years, the landscape of bariatric surgery has been completely twisted. Despite the initial global spread of lap band, scientific community has highlighted its limitations and high failure rate due to unsatisfactory long-term outcomes and occurrence of frequent complications [8].

Therefore, the abandonment of LAGB was worldwide embraced by bariatric surgeons. Conversely, LSG has gained popularity because of its efficacy, safety and reproducibility, becoming an immovable cornerstone of bariatric surgery. LSG allow a rapid effect on weight loss, acting with both restrictive and endocrine mechanisms [22]. Moreover, as reported in literature, the bariatric procedure most revised worldwide is LAGB, often converted to LSG or gastric bypass [23]. Certainly, it should not be neglected that all types of bariatric procedures are at risk of mid- long-term failure.

Even in our experience, bearing in mind the patient’s needs and the gastric banding limitations, we hypothesized that it was time to retire LAGB.

Nevertheless, recently concerns have been raised about worrying weight regain and unsatisfactory outcomes after the different bariatric procedure at long-term follow-up, in particularly regarding LSG [15, 16]. Felsenreich DM et al., showed that 36% of their LSG patients, required revisional surgery within 10-years [23]. Clapp et al. showed that the weight loss failure rate was found to be higher than expected after LSG, and that it increased accordingly to the length of the analysed follow-up period [24]. The procedure, in fact, could require revisional surgery as Re-Sleeve gastrectomy, RYGB or one anastomosis gastric by-pass exposing patients to a further intervention and eventual complications [16, 25, 26]. It is worth to comment that the high absolute number of patients experiencing not satisfying results (insufficient EWL or WR) after LSG are related, on the other hand, to the widespread diffusion of the latter technique among obese patients.

Considering the revision prevalence after other bariatric procedures and the easiness and reversibility of LAGB, we decided to brush-up and retrospectively analyse our previous large series of patients underwent LAGB, focusing on the management of LAGB and analysing potential relationship between post-placement care (band filling quantity) and long-term results and complications [4, 27–34].

After LAGB, successful weight loss depends, in fact, on several factors (e.g., follow-up, band adjustment approach, patient compliance with postoperative recommendations, the band’s pressure–volume characteristics). The heterogeneity in after-care methods employed by the different physicians contributed to the wide range of % EWL reported in literature (31.1–56.7%) [35]. Despite several reports have been published about LAGB anthropometric and surgical outcomes, in literature lacks guidelines and analysis about its postoperative conduction.

The to the best of our knowledge this is the first study analysing the anthropometric and surgical outcomes after LAGB stratifying patients according to the device adjustment.

In details, we observed that minimum band filling (≤ 3 ml) reached a higher rate of % EWL (49.1 ± 11.3 vs 38.2 ± 14.2, *p* < 0.05), a lower BMI (30.3 ± 2.8 vs 35.8 ± 3.7), experiencing also a higher postoperative quality of life (BAROS Score 5.9 ± 1.8 vs 3.8 ± 2.5, *p* < 0.05) at a long-term follow-up. Moreover, the patients with lower band filling (Group 1) complained less episodes of vomiting, epigastric pain and post-prandial reflux and significantly decreased slippage and migration rate (*p* < 0.001 for all parameters). Noteworthy, these patients presented a lower rate of LAGB removal (12 vs 155, *p* < 0.001) and of further bariatric procedure (5 vs 52, *p* < 0.001) with long-lasting (74.3 ± 18.5 vs 52.3 ± 31.6) and increased patient ‘satisfaction.

This data were not surprising and supported the key-role of band calibration and management to achieve satisfying and lasting results in patients with LAGB. It is noteworthy that, the longer the follow-up, the more significant is a 50% EWL because it not only signifies weight loss maintenance but also prevention of the natural weight gain course of the disease.

Conversely, in our series, patients with a hyper-regulation of gastric banding reached an earlier but probably transitory relevant weight loss. This misleading positive result was often related to rapid onset of postoperative dysphagia, nausea and vomiting. Patients, in fact, at the beginning often tolerated these symptoms being satisfied of the increasing weight loss. We found that QoL of these patients improved consensually in an initial phase, demonstrated by a high BAROS Score, despite the onset of symptoms. Only in rare cases they attended, during the earlier period, control visit for a LAGB deflation until they were forced by the high and persistent discomfort of the symptoms. However, considering all the follow-up period, the high QBF was also significantly associated with the postoperative device deflation for the patients’ symptoms intolerance. Twenty patients of Group 1, in fact, experienced a partial deflation vs 100 patients in Group 2 (*p* < 0.0001), while a total deflation occurred in 5 patients of Group 1 and 105 patients of Group 2 (*p* < 0.0001).

Moreover, in accordance with literature data [36] the odds ratio analysis of our data showed that a band overfilling (Group 2) was significantly associated to higher postoperative complications and events. In details, High Band Filling was often correlated to proximal stomach dilatation and to a higher slippage rate (60 vs 1, *p* < 0.001) and migration rate (30 vs 1, *p* < 0.001). The mechanism seemed to be related to the high band pressure determining an over-tightening of the device with the consequent frequent vomiting. The violent and imperious subsequent gastric movements are the possible cause of LAGB slippage [37]. In our series, in case of band migration or slippage, we preferred its prompt removal followed by an eventual bariatric reintervention in a second step, to not underestimate a possible gastric wall erosion or fistula.

Removal of the gastric device was necessary even for patients that complained of persistent slippage-related symptoms, without a specific diagnosis of slippage/migration. This patient’s subset was characterized by band overfilling as well (8 Group 2 vs 3 Group 1).

Regular outpatient visits are largely recommended in obese patients who underwent LAGB, making gradual and minimal increases in the quantity of the band filling until the optimal volume is reached [38]. This latter is determined by the patient’s subjective feeling of good hunger and satiety control without significant restrictive symptoms such as regurgitation, dysphagia, and reflux and minimal device inflation. Several studies demonstrated that the rapid fill of the band, prompted by the patient’s desire to lose weight, is associated to a high complication rate and reduced long-term weight loss, and therefore, has been worldwide discouraged [39]. It is a popular and misleading perception between patients that success of LAGB is closely related to overfilling of band. Similarly, the appearance of lack of satiety and a slight weight loss often lead the surgeon to an earlier adjustment of the band. It is a mistake to assume this aggressive attitude. The current data showed how a gradual and minimum band filling associated with patient compliance and tolerance provides satisfactory long-term results, reducing the incidence of postoperative symptoms.

Flint et al. reported, in their series on 125 LAGB, that the maximum rate of weight loss was reached in the first month when the banding was not inflated [39]. The two third of their cohort reached the optimal LAGB regulation within 1 year from surgery, while as time progressed, the repeated band inflations determined little effect on the rate of weight loss and there was no correlation between the volume in the band and the change in weight [39]. Therefore, it is noteworthy that the pathophysiology of weight loss in patients undergoing LAGB is not strictly related to the band volume but is based on a multifactorial aetiology. Furbetta et al. reported, in fact, excellent anthropometric outcomes on 3566 patients who underwent LAGB reaching a mean of 49, 53.6, and 59.2% EWL at 10, 15, and 20 years, respectively, demonstrating that LAGB can be a highly effective surgical treatment of obesity when a multidisciplinary approach is used [40].

The positive results of our long follow-up series (% EWL of 49.1 ± 11.3, BMI 30.3 ± 2.8 at 74.3 ± 18.5 months follow up, in low band filling Group), might probably prompt to reconsider LAGB “retirement”, as it could be a significant tool among bariatric procedures. Considering, in fact, that even the most adopted LSG is not free from inadequate outcomes and revisional surgery, finding an appropriate and shared management of gastric banding could be an important goal in bariatric surgery. Considering the abovementioned anthropometric outcomes of the current series after LAGB, even if in the selected group with low QBF, the latter values were similar to the ones after LSG (% EWL ranging from 49 to 55%) and lower compared to the anthropometric results after RYGB (% EWL ranging from 56 to 63%) [31, 41–46]. Nevertheless, the Authors are far to presume similar outcomes between LAGB and others more invasive restrictive procedures (i.e., LSG).

Moreover, some Authors argued that the adjustability of the LAGB does not equate with maximization of weight loss, but its advantage is the easy and immediate resolution of symptoms. The onset of symptoms like dysphagia and vomiting, in fact, can be rapidly relieved by an easy deflation of the device, without the need for surgery [3].

Another key point in the surgical outcome after LAGB is certainly the patients’ selection. Several authors identified as positive prognostic factors for postoperative weight loss, the attendance of a high number of outpatients visits and the patients’ compliance [47, 48]. Orlowski et al., investigated this issue in a recent review, concluding that compliance was an independent prognostic factor for weight loss after LAGB in long-term observation and older age was the only factor associated with better compliance [49]. Conversely, opposite results were reached by Leca et al. on 99 LAGBs. They identified, in fact, better anthropometric outcomes in younger age, lower degree of obesity, and lower severity of comorbidities at the time of surgery which were considered predictors of successful weight loss [50, 51].

It is worth to comment that probably the large number of studies demonizing LAGB for the poor consideration on efficacy and complications might has been misinterpreted. As demonstrated in our large series, LAGB is effective even in long-term periods in selected patients that necessitated few regulation and inflation. Therefore, efforts should be made to find predictive selection criteria able to preoperatively identify suitable patients [52].

This study has some limitations to address. First the retrospective manner of the study did not allow any correction of confounding factors (e.g., eventual other risk factors may be present and not measured, prone to recall bias or misclassification bias). In the current series two different LAGB devices were adopted, even if the maximum QBF was the same. Moreover, complications were only recorded if they required clinical intervention (such as re-operation) so might be under-reported.

LAGB is an easy, reversible and reproducible procedure that does not alter the anatomy of the upper gastrointestinal tract. Thanks to its adjustability, LAGB might be full considered among the options in bariatric patients and finding an appropriate handling could have a great impact on the outcomes of this procedure. Therefore, should we really retire the LAGB if more precise outpatient adjustment can be performed and the revision prevalence after other bariatric procedures is increasing? Further prospective studies are needed to address this issue.

## Data Availability

The datasets used and/or analyzed during the current study are available from the Division of General, Mininvasive and Bariatric Surgery, University of Campania “Luigi Vanvitelli”, Naples Italy, on reasonable request.
